# The clinical analysis of 3 patients diagnosed with sporadic Creutzfeldt-Jakob disease: A case report

**DOI:** 10.1097/MD.0000000000048860

**Published:** 2026-05-22

**Authors:** Gulinishahan Abulimiti, Ailiyaer Yasheng, Halidan Jiamaliding, Xiarepa Abudula, Xin Ma, Hasiyeti Yibulaiyin

**Affiliations:** a Department of Neurology, The Second Affiliated Hospital of Xinjiang Medical University, Urumqi, China; b Department of Rehabilitation, Uyghur Medical Hospital of Xinjiang Uyghur Autonomous Region, Urumqi, China; c Department of Surgery and Anesthesiology, The Fourth Affiliated Hospital of Xinjiang Medical University (Xinjiang Hospital of Traditional Chinese Medicine), Urumqi, China; d Xinjiang Uygur Autonomous Region Center for Disease Control and Prevention, Urumqi, China.

**Keywords:** 14-3-3 protein1, case report, cranial MRI, Creutzfeldt–Jakob disease, diagnosis, rapidly progressive dementia

## Abstract

**Rationale:**

Sporadic Creutzfeldt-Jakob disease (sCJD) is a rare and highly fatal neurodegenerative disorder with heterogeneous clinical presentations, making accurate early diagnosis challenging. Furthermore, there is a critical paucity of epidemiological and clinical data regarding sCJD in the Xinjiang region, contributing to frequent misdiagnoses.

**Patient concerns:**

We present 3 patients who exhibited rapidly progressive dementia alongside multifocal neurological signs, including ataxia, extrapyramidal symptoms, and visual disturbances.

**Diagnoses:**

Comprehensive evaluations revealed positive cerebrospinal fluid 14-3-3 protein and the M/M genotype at prion protein gene codon 129 without pathogenic mutations in all cases. Cranial magnetic resonance imaging findings varied from early focal restrictions to typical widespread cortical “ribbon signs,” while electroencephalograms lacked classic periodic sharp-wave complexes. All 3 patients fulfilled the 2017 National CJD Research and Surveillance Unit diagnostic criteria for probable sCJD.

**Interventions:**

The patients received multifaceted symptomatic and supportive treatments, including measures to improve cerebral circulation and metabolism, as well as comprehensive nutritional and neurological support.

**Outcomes:**

Despite targeted treatments, no significant clinical improvement was observed. All patients experienced rapid and fatal disease progression, passing away between 1 month and 1.5 years following hospital discharge.

**Lessons:**

The absence of classic electroencephalogram findings, such as periodic sharp-wave complexes, should not delay an sCJD diagnosis. Recognizing clinical “red flags” and utilizing multimodal diagnostic approaches are essential for early recognition and minimizing misdiagnosis, especially in regions with limited clinical data.

## 1. Introduction

Creutzfeldt-Jakob disease (CJD) is a zoonotic, highly fatal central nervous system disorder caused by abnormal prion proteins, also referred to as cortico-striatal-spinal degeneration or subacute spongiform encephalopathy.^[1]^ CJD exhibits both transmissibility and genetic susceptibility, posing a significant public health threat.^[2]^ Among its subtypes, sporadic CJD (sCJD) is the most prevalent, accounting for approximately 85% of all cases.^[3]^ Disease progression in sCJD is characteristically rapid, with a median survival time from symptom onset to death of only 6 to 12 months.^[4]^The clinical presentation of sCJD is complex and heterogeneous, typically with an insidious onset. Primary symptoms include cognitive decline, myoclonus, ataxia, and behavioral changes.^[5]^ Early diagnosis remains highly challenging owing to the lack of symptom specificity and substantial overlap with other neurological disorders.^[6]^ The current diagnosis of sCJD relies on comprehensive clinical evaluation incorporating electroencephalography (EEG) and cranial magnetic resonance imaging (MRI), whereas definitive confirmation requires brain biopsy to detect the pathological prion protein.^[7]^ However, brain biopsy is rarely performed in clinical practice due to its invasiveness and the associated risk of prion transmission.

In China, sCJD is a rare disease that is frequently misdiagnosed or subject to delayed diagnosis, largely attributable to healthcare providers’ limited familiarity with its characteristic manifestations. The low incidence and inherent diagnostic challenges have resulted in a paucity of clinical data, further hindering physicians’ ability to recognize and manage the disease. These gaps underscore the critical need for case reports and systematic studies to enhance diagnostic precision, elucidate patterns of disease progression, and explore therapeutic strategies. In the present study, we retrospectively analyzed the medical histories, clinical manifestations, imaging findings, and biomarkers of 3 patients with sCJD. Through this approach, we aim to delineate the clinical signature and progression dynamics of sCJD, assess opportunities for earlier detection, and contribute essential evidence toward addressing the clinical data deficiencies in this field. The 3 patients included in this study were diagnosed with probable sCJD upon discharge from the Department of Neurology at the Second Affiliated Hospital of Xinjiang Medical University between February 2019 and December 2021. All patients fulfilled the diagnostic criteria established by the 2017 National CJD Research and Surveillance Unit (NCJDRSU),^[8]^ as presented in Table 1.

**Table 1:** 2017 NCJDRSU diagnostic criteria.

Diagnostic categories	
Clinical manifestation	I: Rapidly progressive cognitive dysfunction Ⅱ: A. myoclonus; B. visual or cerebellar symptoms; C. pyramidal tract or extrapyramidal features; D. inactive mutism
Definite sCJD	Rapidly progressive neurologic symptoms Determined by neuropathology or immunohistochemistry or biochemistry
Probable sCJD	2 of Ⅰ + Ⅱ + PSWCs on EEG. 2 of Ⅰ + Ⅱ + MRI shows abnormal high signal in the basal nucleus or at least 2 abnormal high signals in the cortical area on DWI or FLAIR. 2 of Ⅰ + Ⅱ + CSF14-3-3 protein positive. Progressive neurological symptoms + positive RT-QuIC in CSF or other tissues.
Possible sCJD	2 of Ⅰ + Ⅱ + disease duration < 2 yr

CSF = cerebrospinal fluid, DWI = diffusion-weighted imaging, EEG = electroencephalogram, FLAIR = fluid-attenuated inversion recovery, NCJDRSU = National Creutzfeldt-Jakob disease Research and Surveillance Unit, PSWCs = periodic sharp-wave complexes, RT-QuIC = real-time quaking-induced conversion, sCJD = sporadic Creutzfeldt-Jakob disease.

## 2. Case report

### 2.1. Case 1

The patient, an 81-year-old woman, was admitted to the hospital on February 20, 2019, presenting with sudden-onset left-sided limb weakness accompanied by slurred speech for 27 days, with symptom aggravation over the preceding 16 days. On January 26, 2019, the patient had developed sudden weakness of the left limbs, manifesting as difficulty lifting the left upper limb, inability to hold objects steadily, and dragging of the left lower limb. These symptoms were accompanied by slurred speech and blurred vision, without limb numbness, choking on water intake, or dysphagia. On February 5, the patient’s condition deteriorated further, characterized by drowsiness, leftward lateralization of the body, and choking when drinking water. The patient received treatment at Hainan Hospital; however, no clinical improvement was achieved. On February 20, the patient was admitted to the emergency department of our hospital with a National Institutes of Health Stroke Scale score of 13. Cranial computed tomography (CT) revealed no evidence of hemorrhage. As the thrombolytic time window had been exceeded and thrombolytic therapy was not feasible, the patient was subsequently admitted to the cerebral infarction department. During the disease course, the patient exhibited lethargy, despondency, visual hallucinations, cognitive impairment, increased urinary frequency and urgency, constipation, poor appetite, and expectoration of white sputum. In addition, the patient had experienced a weight loss of approximately 6 to 8 kg over the preceding month.

On admission, the patient’s vital signs were as follows: body temperature 36.5°C, pulse rate 80 beats per minute, respiratory rate 20 breaths per minute, and blood pressure 145/85 mm Hg. Auscultation revealed coarse breath sounds bilaterally. Examination of the heart and abdomen revealed no abnormalities. Neurological examination revealed somnolence, dysarthria, a shallow right nasolabial fold, leftward deviation of the mouth angle, rightward deviation of the tongue on protrusion, a diminished pharyngeal reflex, left upper limb muscle strength of grade 0, left lower limb muscle strength of grade 1, right limb muscle strength of grade 4, and a positive left Babinski sign. Meningeal irritation signs were negative.

### 2.2. Case 2

The patient, a 55-year-old male, was admitted to the hospital on December 7, 2020, due to unresponsiveness and limb stiffness persisting for 1 year, accompanied by worsening weakness of the left limbs over the preceding 2 weeks. One year prior to admission, the patient had developed unresponsiveness without identifiable triggers, manifesting as episodes of driving into obstacles, a dull gaze, and indiscriminate text messaging behavior, accompanied by limb stiffness, which initially went unnoticed. Eight months later, the symptoms intensified, and the patient was admitted to the Hospital of the Sixth Agricultural Division. Cranial MRI revealed multiple punctate infarct foci and diffuse cerebrovascular changes. Following hyperbaric oxygen therapy, the patient was discharged. Seven months later, the patient presented with a dull gaze and memory loss and was admitted to the Xinjiang Military Region General Hospital. Cranial MRI demonstrated ischemic and infarction foci in both cerebral hemispheres. EEG revealed medium-amplitude sharp waves and triphasic waves, particularly prominent in the left occipital region. The diagnoses included: cortical-striatal-spinal degeneration and multiple cerebral infarctions. The patient was treated with antiplatelet, antihypertensive, and lipid-regulating therapies, with unsatisfactory outcomes. Two weeks prior to the current admission, the patient experienced symptom exacerbation characterized by weakness of the left upper and lower limbs, leftward tilting, forward leaning, and a festinating gait during ambulation, frequently resulting in falls. These symptoms were accompanied by urinary and fecal incontinence. The patient was initially admitted to a local hospital, where the diagnosis remained unclear. Subsequent administration of methyldopa tablets led to further clinical deterioration, prompting transfer to our hospital. During the disease course, the patient exhibited marked mental deterioration accompanied by diaphoresis, urinary incontinence, poor sleep quality, choking on water intake, limb tremor, impaired recent memory, and hallucinations.

On admission, the patient’s vital signs included a body temperature of 36.7°C, pulse rate of 64 beats per minute, respiratory rate of 16 breaths per minute, and blood pressure of 125/86 mm Hg. Bilateral breath sounds were present, the heart rate was 64 beats per minute with a regular rhythm, and the abdomen was soft and nontender. Mild bilateral lower limb edema was noted, and no significant pathological signs were observed. Neurological examination revealed clear consciousness with unresponsiveness, a dull facial expression, a shallow left nasolabial fold, increased muscle tone in all limbs, left upper limb distal muscle strength of grade 3 and proximal grade 4, left lower limb muscle strength of grade 4, right limb muscle strength of grade 5, equivocal pathological signs on the left side, and negative meningeal irritation signs.

### 2.3. Case 3

The patient, a 67-year-old female, was admitted to the hospital on December 27, 2021, presenting with a 1-month history of decreased vision and slowed responsiveness, exacerbated by unsteady gait for 1 week. In early November 2021, the patient developed decreased vision, slowed responsiveness, and diminished facial expression without identifiable precipitating factors. She denied headache, nausea, and vomiting. The patient was admitted to Qitai County People’s Hospital on November 17, 2021, where cranial CT suggested multilobar cerebral infarction, white matter degeneration, and mild cerebral atrophy. A diagnosis of cataract was made and corresponding treatment was administered. Although vision improved slightly, symptoms worsened 1 week prior to the current admission, with the patient reporting limb tremor and unsteady gait. Consequently, the patient returned to the hospital, where further diagnostic imaging revealed disease progression. At the time of presentation, the patient was coherent and alert, denied fever and cough, and reported poor appetite with adequate sleep. On examination, the patient’s vital signs were as follows: temperature 36.6°C, pulse 68 beats per minute, respiratory rate 17 breaths per minute, and blood pressure 101/67 mm Hg. Neurological examination revealed a dull expression, slow speech, bradykinesia, unsteady gait, and impaired calculation ability. Limb muscle strength was normal, muscle tone was increased, and swallowing function was preserved (Table 2).

**Table 2:** Patient demographics.

Case number	Age	Gender	Occupation	Medical history	Date of onset	Date of diagnosis	Date of death
1	81	Female	Retiree	High blood pressure, diabetes, and coronary heart disease	January 26, 2019	March 2019	April 2019
2	55	Male	Self-employed	High blood pressure	December 2019	December 2020	June 2021
3	67	Female	Farmer	Coronary heart disease	Early November 2021	December 2021	September 2022

## 3. Analysis of clinical data

### 3.1. Symptoms and signs

The mode of onset was subacute in 2 cases and chronic in 1 case. The initial presenting symptoms were limb weakness in 1 case, unresponsiveness in 1 case, and decreased visual acuity in 1 case. The principal clinical manifestations included progressive dementia in all 3 cases, behavioral disturbances in 1 case, ataxia in 1 case, extrapyramidal signs in 2 cases, pyramidal tract signs in 2 cases, and vision loss in 2 cases (Table 3).

**Table 3:** Clinical manifestations and progression.

Case number	First symptoms	Main clinical symptoms	Neurological examination			MMSE scores	Course of the disease
			Pyramidal signs	Extrapyramidal signs	Cerebellar signs		
1	Limb weakness	Have difficulty speaking, visual defects, cognitive impairment	√			15	27 d
2	Slow reaction	Mental behavioral abnormalities, rigidity, limb weakness	√	√		23	1 yr
3	Visual defects	Unsteady walking, slow reaction, body tremor		√	√	11	1 mo

MMSE = Mini-Mental State Examination.

### 3.2. Ancillary investigations

#### 3.2.1. Blood test results

Prion protein gene (PRNP) gene sequence analysis of the 3 patients yielded the following results: comparison with the reference sequence revealed no pathogenic mutations; the amino acid polymorphism at codon 129 was of the M/M-type; and the amino acid polymorphism at codon 219 was of the E/E-type (Table 4).

**Table 4:** Laboratory and ancillary examination results.

Case number	Cranial MRI (DWI)	EEG	14-3-3 protein	PRNP gene polymorphisms	
				129 amino acids	219 amino acids
1	High brain signal in the right hemisphere	Diffuse slow waveƟ waves and a few short bursts of δ-wave issuance in both cerebral hemispheres	(-)	M/M-type	E/E-type
2	Cerebral impotence	Mild widespread abnormal EEG	(-)	M/M-type	E/E-type
3	High signal in bilateral frontotemporo-parieto-occipital lobes, right basal ganglia region	Abnormal EEG with triphasic wave emission, more on the right side.	(-)	M/M-type	E/E-type

DWI = diffusion-weighted imaging, EEG = electroencephalogram, PRNP = prion protein gene, MRI = magnetic resonance imaging.

#### 3.2.2. Cerebrospinal fluid results

All 3 patients underwent lumbar puncture, and cerebrospinal fluid was tested for the 14-3-3 protein. The 14-3-3 protein assay was positive (Western blot method) in all 3 patients (Table 4).

#### 3.2.3. Cranial MRI findings

**Figure 1.**
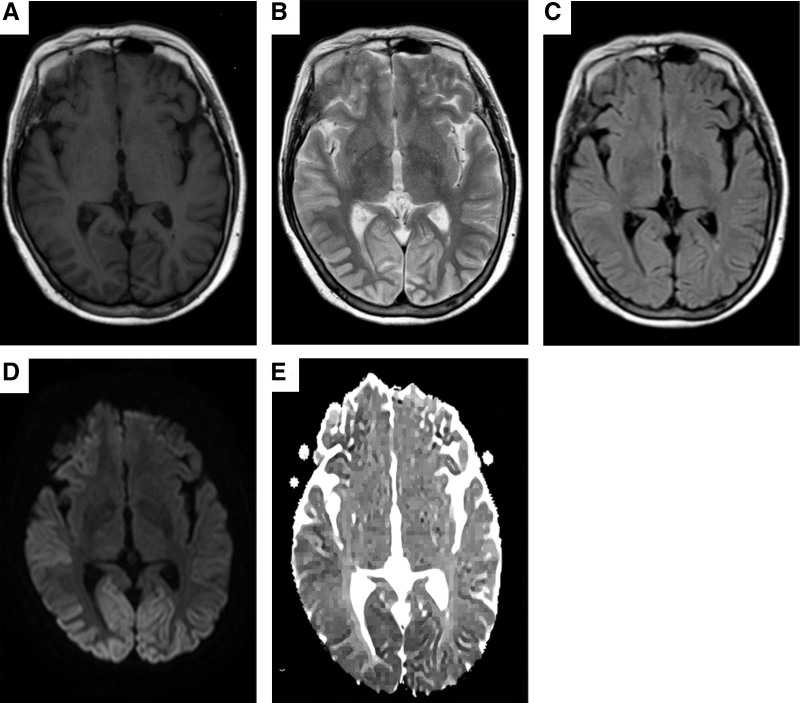
Cranial MRI of case 3. The cranial MRI of the 3rd patient is shown in A to E. (A) Transverse axial T1WI showed no obvious abnormal signal. (B) Transverse axial T2WI showed high signal in bilateral occipital, right frontal and temporal lobes. (C) Transverse axial FLAIR shows slightly high signal in the right temporal lobe. (D) Transverse axial DWI sequence showing high signal in the bilateral occipital, right frontal and temporal lobes, typical of ribbon sign. (E): ADC shows low signal in the area corresponding to the DWI. DWI = diffusion-weighted imaging, FLAIR = fluid-attenuated inversion recovery.

In the 1st patient, atypical signal intensity was observed in the right cerebral hemisphere gyrus. In the 2nd patient, minor cerebral white matter demyelination and brain atrophy were detected, with no abnormality identified on diffusion-weighted imaging (DWI). In the 3rd patient, abnormal signals were detected in the bilateral occipital, parietal, frontal, and temporal lobes as well as the right basal ganglia region, manifesting as laminar, gyrus-like abnormalities (Fig. 1).

#### 3.2.4. Electroencephalogram findings

**Figure 2.**
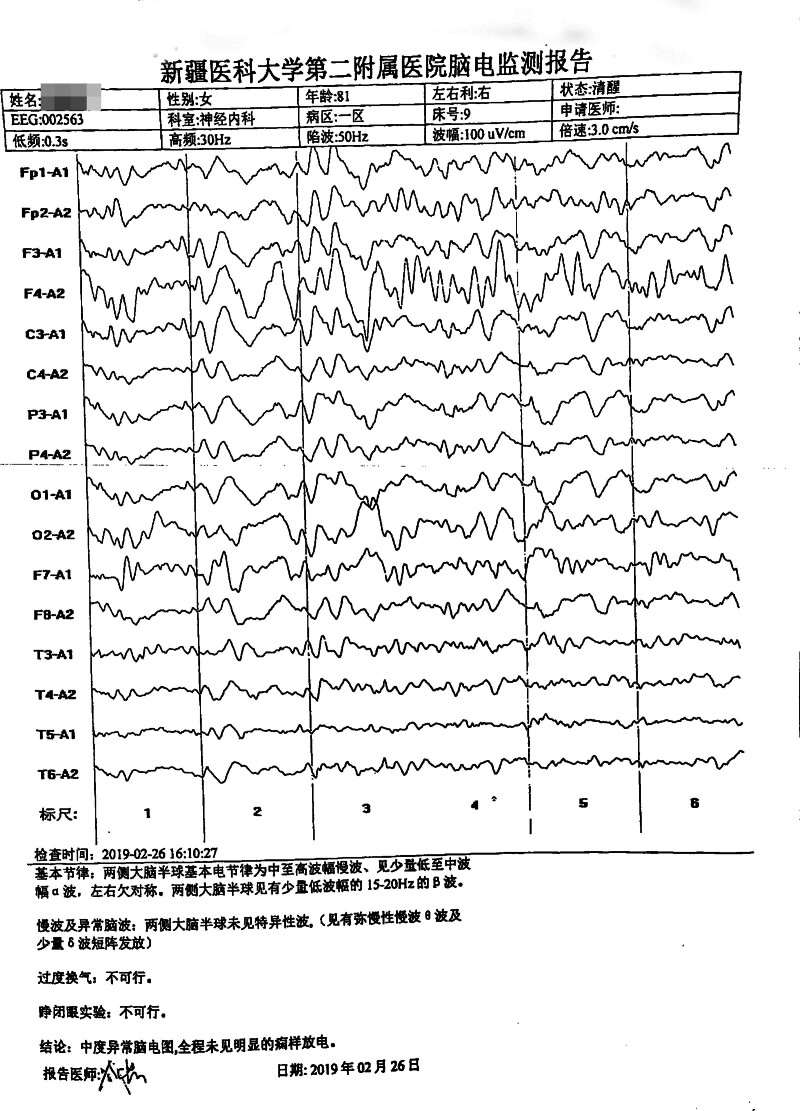
EEG of case 1. The figure shows the EEG of case 1: diffuse slow-wave and theta-wave in both cerebral hemispheres and some delta wave short burst issuance. EEG = electroencephalogram.

**Figure 3.**
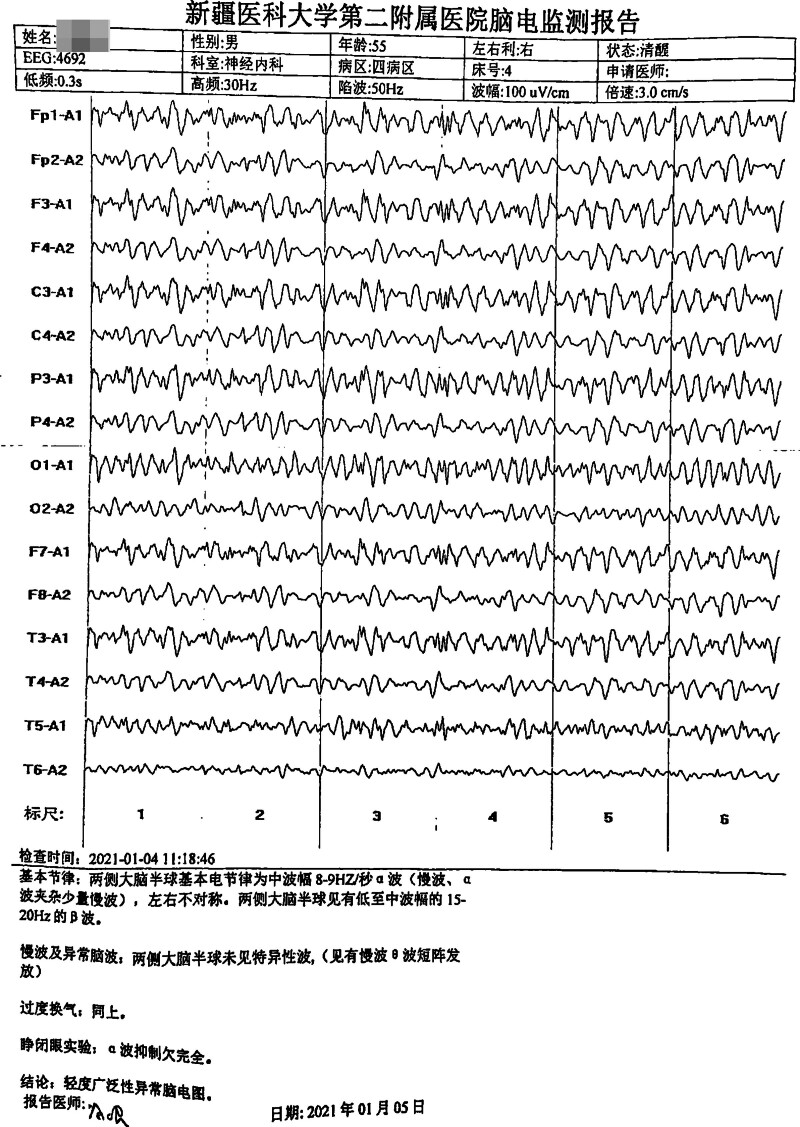
EEG of case 2. The figure shows the EEG of case 2: mild widespread abnormalities. EEG = electroencephalogram.

**Figure 4.**
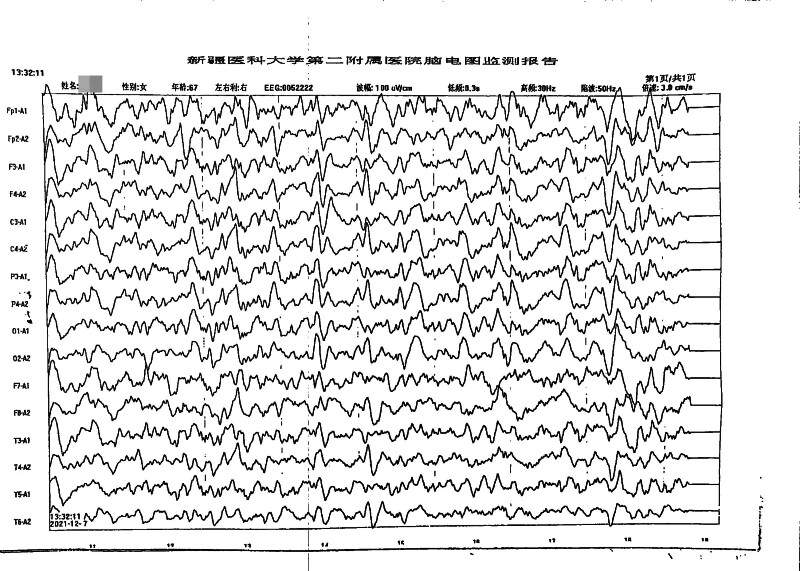
EEG of case 3. The figure shows the EEG of case 3: a typical triphasic wave is seen (most pronounced in the area indicated by the arrow). EEG = electroencephalogram.

In case 1, a moderately abnormal EEG was observed; however, no significant epileptiform discharges were identified. The EEG revealed diffuse slow-wave and theta-wave activity, accompanied by occasional delta waves and short bursts of discharges over both cerebral hemispheres (Fig. 2). In case 2, the EEG demonstrated mild diffuse abnormalities characterized by short bursts of slow-wave and theta-wave activity (Fig. 3). In case 3, the 24-hour EEG revealed abnormal activity characterized by triphasic wave discharges, which were more prominent over the right hemisphere (Fig. 4).

### 3.3. Disease progression

All 3 patients were diagnosed with probable sCJD based on comprehensive case characterization. Upon admission, multifaceted interventions were implemented, including measures to improve cerebral circulation, promote cerebral metabolism, and provide nutritional and neurological support along with symptomatic therapy. Despite targeted treatment, no significant clinical improvement was observed during the hospitalization period. All 3 patients unfortunately died from disease progression within a period ranging from 1 month to 1½ years following discharge, underscoring the high lethality of sCJD and the limitations of currently available treatments.

Below is the professionally edited version of your Discussion and Conclusion sections. All abbreviations, reference numbers, and technical terminology have been preserved, while grammar, syntax, word choice, and overall academic tone have been refined.

## 4. Discussion

### 4.1. Clinical manifestations

sCJD represents the most prevalent form of human prion disease.^[9]^ It predominantly affects middle-aged and elderly populations, with peak incidence occurring between 50 and 80 years of age and a mean survival duration of approximately 6 months.^[10]^ The 3 patients in the present study were aged 81, 55, and 67 years, respectively, and their ages at symptom onset were consistent with those reported in both national and international literature.^[10]^ Cognitive impairment constitutes the cardinal clinical manifestation of sCJD, typically presenting as rapidly progressive dementia (RPD),^[11]^ which is often the earliest and most prominent indicator of the disease. RPD is characterized by either an abrupt or insidious onset, with patients experiencing a precipitous decline in cognitive function over a relatively brief period.^[12]^ All 3 patients in this study exhibited features consistent with RPD, in addition to behavioral disturbances, ataxia, extrapyramidal symptoms, and vision loss – findings that are concordant with those of other studies conducted in China.^[13]^ sCJD has the capacity to affect multiple brain regions, with a particular predilection for the cerebral cortex. Consequently, its clinical presentation is complex and highly variable, frequently mimicking a broad spectrum of other neurological and psychiatric disorders.^[14,15]^ Approximately 30% of patients initially present with atypical symptoms, including dizziness and sleep disturbances.^[16]^ The initial symptoms in the present study were heterogeneous, encompassing limb weakness (1 case), unresponsiveness (1 case), and vision loss (1 case). The principal clinical signs and symptoms included progressive dementia (3 cases), behavioral abnormalities (1 case), cerebellar ataxia (1 case), extrapyramidal signs (2 cases), pyramidal tract signs (2 cases), and vision loss (2 cases). According to the “China Diagnostic Guidelines for Creutzfeldt-Jakob Disease 2021,”^[16]^ a comprehensive evaluation encompassing meticulous assessment of the cortical, cerebellar, pyramidal, and extrapyramidal systems, in conjunction with a detailed physical examination, is imperative in patients with suspected CJD. The 3 patients in this study were ultimately diagnosed with probable sCJD following a thorough history-taking and physical examination that revealed cognitive deterioration, supplemented by a series of ancillary investigations. Notably, traditional diagnostic methods that rely primarily on clinical assessment have inherent limitations in terms of accuracy and specificity.^[17]^

### 4.2. Differential diagnosis

A primary differential diagnosis considered was autoimmune encephalitis, which can present with rapid cognitive decline and psychiatric symptoms. However, the negative results for relevant autoantibodies in both serum and cerebrospinal fluid (CSF), combined with the absence of paraneoplastic evidence, effectively ruled out this diagnosis.

Viral encephalitis was also considered but deemed unlikely. The patients lacked typical infectious prodromes such as fever and headache, showed no signs of meningeal irritation, and had normal routine and biochemical CSF analyses – findings inconsistent with the pleocytosis and elevated protein levels characteristic of viral encephalitis.

Toxic-metabolic encephalopathies, including Hashimoto encephalopathy, were excluded based on normal thyroid function tests and negative thyroid-related autoantibodies in all patients. Other toxic or metabolic etiologies were considered improbable given the absence of toxin exposure history or severe systemic organ dysfunction.

Vascular dementia was distinguished by its characteristic stepwise progression, which contrasts with the continuous and diffuse deterioration observed in our patients. The MRI findings – particularly the typical cortical ribboning on DWI – were inconsistent with the multiple infarct pattern seen in vascular dementia.

Following this systematic exclusion process, the diagnosis converged on probable sCJD. This conclusion was established in strict accordance with the 2017 NCJDRSU diagnostic criteria. Specifically, all patients fulfilled the criteria by presenting with: RPD; at least 2 core clinical signs (myoclonus, visual or cerebellar dysfunction, and pyramidal/extrapyramidal signs); and a positive supportive laboratory finding, namely elevated 14-3-3 protein in CSF or typical high-signal abnormalities on DWI/fluid-attenuated inversion recovery (FLAIR) MRI. As genetic CJD was excluded by negative PRNP gene mutation analysis and there was no history of iatrogenic exposure, probable sCJD represents the most accurate clinical diagnosis for this cohort (as shown in Table 5).

**Table 5:** Fulfillment of 2017 NCJDRSU diagnostic criteria for probable sCJD in 3 cases.

Diagnostic component	Case 1	Case 2	Case 3	NCJDRSU criterion
Rapidly progressive dementia	✓	✓	✓	Required (I)
Myoclonus	–	–	–	≥2 of IIA–IID required
Visual/cerebellar signs	Visual	–	Cerebellar	
Pyramidal/extrapyramidal signs	–	Pyramidal	Extrapyramidal	
MRI (DWI/FLAIR)	Cortical ribboning (right hemisphere)	No typical findings	Cortical ribboning (bilateral, multifocal)	Supportive (IIIA)
EEG	No PSWCs	No PSWCs	No PSWCs	Supportive (IIIB)
CSF 14-3-3 protein	–	–	–	Supportive (IIIC)
PRNP codon 129	M/M	M/M	M/M	–
Disease duration	<2 yr	<2 yr	<2 yr	Required for some criteria
Criteria met	I + ≥2 clinical + IIIA	I + ≥2 clinical + duration < 2y	I + ≥2 clinical + IIIA	
Final diagnosis	Probable sCJD	Probable sCJD	Probable sCJD	

All 3 cases met the 2017 NCJDRSU criteria for probable sCJD. Case 1 and case 3 fulfilled the criterion of RPD + ≥2 clinical features + typical MRI findings (cortical ribboning on DWI). Case 2, despite lacking typical MRI abnormalities, met the criterion of RPD + ≥2 clinical features + disease duration < 2 years. None of the cases showed periodic sharp-wave complexes (PSWCs) on EEG or positive CSF 14-3-3 protein, but these are not mandatory for the diagnosis of probable sCJD.

CSF = cerebrospinal fluid, DWI = diffusion-weighted imaging, EEG = electroencephalogram, FLAIR = fluid-attenuated inversion recovery, MRI = magnetic resonance imaging, NCJDRSU = National Creutzfeldt-Jakob disease Research and Surveillance Unit, PRNP = prion protein gene, PSWCs = periodic sharp-wave complexes, RPD = rapidly progressive dementia, sCJD = sporadic Creutzfeldt-Jakob disease.

Following this process of systematic exclusion, the diagnosis converged on probable sCJD. This conclusion was established in strict accordance with the 2018 diagnostic criteria from the Centers for Disease Control and Prevention. Specifically, all patients fulfilled the criteria by presenting with: RPD; at least 2 core clinical signs (myoclonus, visual or cerebellar dysfunction, and pyramidal/extrapyramidal signs); and a positive supportive laboratory finding, namely elevated 14-3-3 protein in the CSF or typical high-signal abnormalities on DWI/FLAIR MRI. As genetic CJD was excluded by negative PRNP gene mutation analysis and there was no history of iatrogenic exposure, probable sCJD represents the most accurate clinical diagnosis for this cohort.

### 4.3. Neuroimaging

In the radiological assessment of sCJD, cranial MRI – particularly DWI – serves as a crucial noninvasive diagnostic tool. Typical manifestations include cortical “ribbon sign” or abnormal hyperintensity in the basal ganglia, with reported sensitivity ranging from 67% to 91% and specificity of 97%.^[18,19]^ Recent advances in MRI technology have further enhanced sensitivity to as high as 99%.^[20]^ Notably, abnormal DWI signals may occasionally precede clinical symptoms, EEG abnormalities, and CSF changes,^[21]^ underscoring its value in early detection.

In the present study, cranial MRI findings varied considerably among the 3 sCJD patients, reflecting different disease stages and phenotypic presentations:

Case 1: Follow-up MRI on admission revealed new focal diffusion restriction and FLAIR hyperintensity in the gyral regions of the right cerebral hemisphere, consistent with early cortical “ribbon sign.” This unilateral, relatively localized pattern suggests early-stage disease with pathological prion protein accumulation predominantly affecting 1 hemisphere.
Case 2: MRI demonstrated brain atrophy without typical DWI or FLAIR hyperintensity. While isolated atrophy lacks specificity for sCJD diagnosis, this case fulfilled the probable sCJD criteria based on the combination of rapidly progressive dementia, at least 2 core clinical features (pyramidal and extrapyramidal signs), and disease duration <2 years (2017 NCJDRSU criterion IV). The absence of typical MRI findings may represent an atypical early presentation or a phenotypic variant with predominant neuronal loss rather than spongiform change.
Case 3: MRI revealed extensive and confluent DWI/FLAIR hyperintensity involving bilateral occipital, parietal, frontal, and temporal cortices, as well as the right basal ganglia – a typical widespread cortical “ribbon sign.” This extensive lesion burden is consistent with intermediate-to-advanced disease stage. Notably, despite the widespread MRI abnormalities suggesting more advanced pathology, this patient this patient survived for approximately 10 months, which is longer than the median survival reported in sCJD cohorts with similar imaging patterns.^[22]^ This observation suggests that the extent of MRI abnormalities, while indicative of disease stage, may not directly correlate with survival duration in all cases, possibly reflecting individual variability in disease progression or compensatory mechanisms.

### 4.4. Electroencephalogram (EEG)

Electroencephalography serves as an important noninvasive diagnostic tool for sCJD.^[22]^. Periodic sharp-wave complexes (PSWCs) – defined by periodic sharp waves, polyspikes, and slow-wave complexes with 0.5 to 2 second periodicity against diffuse low-voltage slow waves^[23]^represent the most characteristic EEG feature of sCJD, with high specificity but variable sensitivity (38.2%–68.75%).^[24]^ Critically, PSWCs typically emerge only in intermediate-to-advanced disease stages, while early-stage EEG findings are often limited to nonspecific diffuse slow-wave activity.

In the present study, none of the 3 patients exhibited typical PSWCs, which warrants careful interpretation in the context of disease stage and diagnostic implications:

Case 1: EEG demonstrated moderately abnormal findings with increased diffuse slow waves (predominantly theta activity) and brief delta wave bursts. This nonspecific pattern is consistent with early cortical metabolic slowing and neuronal dysfunction, representing the typical early-stage EEG presentation of sCJD before PSWCs develop.
Case 2: EEG showed mild abnormalities without typical sCJD features or PSWCs. This may reflect: disease not yet reaching the threshold for characteristic EEG changes, an atypical sCJD subtype with less prominent EEG abnormalities, or early disease stage. The absence of PSWCs in this case underscores that EEG findings must be interpreted alongside other diagnostic criteria – this patient fulfilled probable sCJD criteria based on clinical features and disease duration rather than EEG or MRI findings.
Case 3: EEG revealed triphasic waves without typical PSWCs. While triphasic waves are nonspecific and can occur in various metabolic encephalopathies, their presence in the context of rapidly progressive dementia and extensive MRI abnormalities supports severe cerebral dysfunction consistent with intermediate-stage sCJD. The absence of PSWCs despite advanced imaging findings may indicate: examination performed before PSWC emergence, phenotypic variability in EEG manifestations, or a disease subtype less likely to develop PSWCs.
Early versus late EEG patterns: The temporal evolution of EEG abnormalities in sCJD typically follows a predictable sequence: early stages show nonspecific diffuse slowing (theta/delta activity), intermediate stages may develop PSWCs (usually 8–12 weeks after symptom onset), and late stages demonstrate progressive background suppression with eventual loss of PSWCs. Our cases, lacking PSWCs, likely represent early-to-intermediate disease stages or phenotypic variants. Studies indicate that serial EEG monitoring increases PSWC detection rates,^[25]^ suggesting that single negative EEG examinations should not exclude sCJD diagnosis.
Diagnostic implications: The absence of PSWCs in all 3 cases highlights several important points: PSWCs are not mandatory for probable sCJD diagnosis under 2017 NCJDRSU criteria, early-stage sCJD frequently presents with nonspecific EEG findings, and comprehensive diagnostic assessment requires integration of clinical features, MRI findings, CSF biomarkers, and disease progression patterns. The variability in EEG presentations across our cases – from mild abnormalities (case 2) to triphasic waves (case 3) – reflects the heterogeneity of sCJD phenotypes and underscores the importance of multimodal diagnostic approaches.

### 4.5. Cerebrospinal fluid 14-3-3 protein

The 14-3-3 protein is a widely utilized CSF biomarker that facilitates the early diagnosis of sCJD. It is released into the CSF in response to neuronal damage. Nevertheless, the 14-3-3 protein is not exclusively associated with sCJD; elevated levels have also been observed in cases of stroke.^[26]^ In the presence of inflammation, paraneoplastic syndromes, tumors, and epilepsy, false-positive results may occur.^[27]^ Therefore, a comprehensive judgment integrating clinical manifestations and other investigations is necessary. Studies have demonstrated that the sensitivity of the 14-3-3 protein in diagnosing sCJD ranges from 85% to 95%, with a specificity of 74% to 96%.^[27]^ In patients diagnosed with sCJD, levels of the 14-3-3 protein are typically elevated over time, whereas in patients with acute neuronal injury, these levels decline as clinical recovery occurs. Consequently, the 14-3-3 protein can be utilized not only for diagnostic purposes but also for monitoring disease progression and evaluating therapeutic efficacy. However, it should be noted that the 14-3-3 protein may also yield positive results in other neurodegenerative diseases, such as Alzheimer disease (AD) and Parkinson disease.^[28]^ Its diagnostic value must therefore be interpreted with considerable caution, and a multimodal approach is required to enhance the accuracy of results. In the present study, all 3 patients demonstrated positive CSF 14-3-3 protein test results. These patients exhibited a rapidly progressive clinical course, with symptoms that could not be fully accounted for by extrapyramidal disorders or AD alone. The diagnosis was further corroborated by the results of additional ancillary investigations.

### 4.6. PRNP gene testing

The detection of PRNP gene mutations is valuable for distinguishing genetic from sCJD.^[29]^ While pathogenic mutations cause genetic CJD through aberrant prion proteins folding,^[30]^ polymorphisms at specific codons influence sCJD susceptibility without directly causing disease. In the Chinese Han population, M/M homozygosity at codon 129 (98%) and E/E at codon 219 (94.84%) are highly prevalent^[31,32]^and associated with increased sCJD risk.

All 3 patients were Han Chinese with M/M-129 and E/E-219 genotypes, consistent with the high-risk profile. Notably, no pathogenic PRNP mutations were detected, confirming the sporadic nature of disease. This genotype distribution may contribute to sCJD susceptibility in this population, though the precise mechanism remains unclear.

### 4.7. Clinical learning points and red flags for early recognition

Our findings highlight critical clinical learning points for early sCJD recognition. Based on our case series and literature review, we propose the following “red flags” that should prompt immediate consideration of sCJD:

#### 4.7.1. Primary red flags

(1) RPD: Cognitive decline progressing over weeks to months (typically < 2 years), rather than the gradual deterioration seen in AD. In our series, all 3 patients demonstrated marked functional decline within 3 to 6 months of symptom onset.
(2) Multifocal neurological signs: The simultaneous or sequential appearance of at least two of the following: myoclonus (cases 1 and 3), cerebellar ataxia (all 3 cases), visual disturbances (case 3), pyramidal signs (cases 2 and 3), or extrapyramidal symptoms (cases 1 and 2). The combination of these signs in a rapidly progressive course is highly suggestive of sCJD.
(3) Relentless clinical trajectory: Unlike stroke or other acute neurological events that stabilize or improve, sCJD demonstrates continuous deterioration without plateaus. This pattern was evident in all our cases and distinguishes sCJD from many mimics.

#### 4.7.2. Secondary red flags

(1) Psychiatric symptoms (depression, anxiety, and behavioral changes) preceding or accompanying cognitive decline, particularly in younger patients
(2) Disproportionate functional impairment relative to initial cognitive testing
(3) Lack of response to standard treatments for presumed diagnoses (e.g., antidepressants and antipsychotics)

#### 4.7.3. Common diagnostic pitfalls

A critical learning point from our cases and reported misdiagnoses is the danger of “anchoring bias” – fixating on a single prominent symptom (such as visual disturbance in case 3 or ataxia in case 1) while overlooking the atypical velocity of progression. Clinicians must resist the temptation to attribute symptoms to common conditions (depression, stroke, and AD) without 1st assessing the tempo of decline. A key question to ask is: “Has this patient deteriorated more rapidly than expected for the presumed diagnosis?”

#### 4.7.4. Actionable Clinical Approach

When red flags are present, clinicians should: Expedite brain MRI with DWI/FLAIR sequences (not routine CT); obtain CSF for 14-3-3, *t*-tau, and real-time quaking-induced conversion analysis; perform serial EEG if initial study is nondiagnostic; and avoid prolonged empirical trials of treatments for alternative diagnoses.

This proactive approach, triggered by recognition of these red flags, is essential for minimizing diagnostic delays – particularly in resource-limited settings where advanced tests may be unavailable. Early recognition allows for timely family counseling, infection control measures, and avoidance of unnecessary invasive procedures.

## 5. Conclusion

This study aims to elucidate the complexities of sCJD through a comprehensive review and analysis of 3 cases with a high probability of the disease. The objectives of this study are 2-fold: to delineate the diagnostic challenges associated with sCJD; and to highlight key factors that facilitate its diagnosis. Our analysis reveals the nonspecificity of the clinical manifestations of sCJD and the rapidity of disease progression, both of which can lead to diagnostic confusion and a consequently high rate of misdiagnosis. The paucity of epidemiological data and the limited dissemination of knowledge regarding the disease have contributed to insufficient patient awareness and, in some instances, a lack of familiarity among medical professionals. This study aims to address these deficiencies by meticulously examining the diagnostic process, disease progression, and treatment strategies in 3 cases of sCJD. By doing so, we hope to provide clinicians with a comprehensive and practical diagnostic reference, thereby enhancing their awareness of and vigilance toward sCJD. Concurrently, we disseminate these findings in the form of a case report to address the knowledge gap in disease epidemiology and provide substantive support for future research initiatives.

A systematic literature review of major databases (China National Knowledge Infrastructure and Wanfang) using “CJD” and “prion disease” as search terms reveals a critical knowledge gap in Xinjiang. Only 1 case report exists,^[33]^ documenting 3 probable sCJD patients initially misdiagnosed as vascular dementia, encephalitis, and anxiety disorder. This scarcity of regional data – contrasting sharply with other Chinese regions – highlights deficiencies in both epidemiological surveillance and clinical awareness.

Xinjiang unique position stems from several factors. First, epidemiological void: absence of systematic surveillance makes it impossible to determine whether sCJD incidence differs from national averages or is underestimated due to diagnostic challenges. Second, distinctive misdiagnosis patterns: the documented misdiagnoses mirror our cases (stroke, depression, and cerebellar ataxia) and suggest region-specific diagnostic barriers reflecting limited clinical exposure, inadequate access to specialized facilities (real-time quaking-induced conversion unavailable in most Xinjiang hospitals), and tendency to attribute symptoms to more common conditions. Third, geographic challenges: Xinjiang vast territory (1.66 million km^2^) creates obstacles for timely diagnosis, with patients traveling hundreds of kilometers to tertiary centers, prolonging intervals between symptom onset and specialized evaluation.

Our study addresses these gaps by providing the 1st detailed clinicopathological analysis of probable sCJD cases in Xinjiang with comprehensive diagnostic workup. Beyond documenting presentations, we identify region-specific diagnostic pitfalls and propose strategies to improve recognition in resource-limited settings, with implications for other underserved regions facing similar barriers.

In summary, our study has not only deepened the understanding of the clinical features, diagnostic methods, and prognosis of sCJD but has also contributed to enhancing diagnostic accuracy, rectifying diagnostic inconsistencies, and promoting awareness of the disease. In the future, we will continue to explore emerging therapeutic approaches aimed at intervening in the disease process at the etiologic level and providing more effective treatment strategies for patients.

## Author contributions

**Data curation:** Halidan Jiamaliding, Xiarepa Abudula, Xin Ma

**Investigation:** Halidan Jiamaliding, Xiarepa Abudula, Xin Ma

**Supervision:** Ailiyaer Yasheng, Hasiyeti Yibulaiyin

**Validation:** Ailiyaer Yasheng, Hasiyeti Yibulaiyin

**Writing – original draft:** Gulinishahan Abulimiti

**Writing – review & editing:** Gulinishahan Abulimiti, Ailiyaer Yasheng, Hasiyeti Yibulaiyin

## References

[1] De Melo ASLF Lima JLD Malta MCS. The role of microglia in prion diseases and possible therapeutic targets: a literature review. Prion. 2021;15:191–206. 34751640 10.1080/19336896.2021.1991771 PMC8583147

[2] Fayolle M Lehmann S Delaby C. Comparison of cerebrospinal fluid tau, ptau(181), synuclein, and 14-3-3 for the detection of Creutzfeldt-Jakob disease in clinical practice. J Neural Transm (Vienna). 2022;129:133–9. 35041062 10.1007/s00702-021-02443-8

[3] Langlands G Mackenzie J Graham C. Non-white cases of sporadic Creutzfeldt-Jakob disease: a 28 year review of United Kingdom National Surveillance Data. J Neurol Sci. 2021;424:117416. 33839436 10.1016/j.jns.2021.117416

[4] Zerr I Parchi P. Sporadic Creutzfeldt-Jakob disease. Handb Clin Neurol. 2018;153:155–74. 29887134 10.1016/B978-0-444-63945-5.00009-X

[5] Geschwind MD Murray K. Differential diagnosis with other rapid progressive dementias in human prion diseases. Handb Clin Neurol. 2018;153:371–97. 29887146 10.1016/B978-0-444-63945-5.00020-9

[6] Lee K Park DG Kim MS An YS Yoon JH. Dual-phase 18 F-FP-CIT PET in 2 different clinical phenotypes of sporadic Creutzfeldt-Jakob Disease. Clin Nucl Med. 2022;47:e548–9. 35439185 10.1097/RLU.0000000000004240

[7] Duan S Yang J Cui Z. Seed amplification assay of nasal swab extracts for accurate and non-invasive molecular diagnosis of neurodegenerative diseases. Transl Neurodegener. 2023;12:13. 36922862 10.1186/s40035-023-00345-1 PMC10017346

[8] Saraceno L Ricigliano VAG Cavalli M. Sporadic MM-1 type Creutzfeldt-Jakob disease with hemiballic presentation and no cognitive impairment until death: how new NCJDRSU diagnostic criteria may allow early diagnosis. Front Neurol. 2018;9:739. 30233486 10.3389/fneur.2018.00739 PMC6134320

[9] Cohen D Kutluay E Edwards J Peltier A Beydoun A. Sporadic Creutzfeldt-Jakob disease presenting with nonconvulsive status epilepticus. Epilepsy Behav. 2004;5:792–6. 15380138 10.1016/j.yebeh.2004.06.019

[10] Huang J Cohen M Safar J Auchus AP. Variably protease-sensitive prionopathy in a middle-aged man with rapidly progressive dementia. Cogn Behav Neurol. 2021;34:220–5. 34473674 10.1097/WNN.0000000000000276 PMC8803003

[11] Karamujić-Čomić H Rozemuller AJM Ikram MA Van Duijn CM. First participant diagnosed with Creutzfeldt-Jakob disease in the population-based Rotterdam Study was classified with mild cognitive impairment. BMJ Case Rep. 2021;14:e235509. 10.1136/bcr-2020-235509 PMC8009210 33782059

[12] Abu-Rumeileh S Parchi P. Cerebrospinal fluid and blood neurofilament light chain protein in prion disease and other rapidly progressive dementias: current state of the art. Front Neurosci. 2021;15:648743. 33776643 10.3389/fnins.2021.648743 PMC7994519

[13] Zhang Jin ML Haohao K. Electroencephalographic studies on sporadic Creutzfeldt-Jakob Disease. Apoplexy Nervous Dis. 2023;40:34–8.

[14] Mead S Rudge P. CJD mimics and chameleons. Pract Neurol. 2017;17:113–21. 28153848 10.1136/practneurol-2016-001571 PMC5520355

[15] Chen Y Xing XW Zhang JT. Autoimmune encephalitis mimicking sporadic Creutzfeldt-Jakob disease: a retrospective study. J Neuroimmunol. 2016;295–296:1–8. 10.1016/j.jneuroim.2016.03.012 27235341

[16] Neuroinfectious Diseases and Cerebrospinal Fluid Cytology, Neurology Branch, Chinese Medical Association. Chinese guidelines for diagnosis of Creutzfeldt-Jakob disease 2021. Zhonghua Shen Jing Jing Shen Ke Za Zhi. 2022;55:1215–24.

[17] Kwon GT Kwon MS. Diagnostic challenge of rapidly progressing sporadic Creutzfeldt-Jakob disease. BMJ Case Rep. 2019;12:e230535. 10.1136/bcr-2019-230535 PMC6768346 31551319

[18] Meissner B Kallenberg K Sanchez-Juan P. MRI lesion profiles in sporadic Creutzfeldt-Jakob disease. Neurology. 2009;72:1994–2001. 19506221 10.1212/WNL.0b013e3181a96e5d

[19] Park HY Kim M Suh CH Kim SY Shim WH Kim SJ. Diagnostic value of diffusion-weighted brain magnetic resonance imaging in patients with sporadic Creutzfeldt-Jakob disease: a systematic review and meta-analysis. Eur Radiol. 2021;31:9073–85. 33982159 10.1007/s00330-021-08031-4

[20] Jesuthasan A Sequeira D Hyare H. Assessing initial MRI reports for suspected CJD patients. J Neurol. 2022;269:4452–8. 35362733 10.1007/s00415-022-11087-x PMC9293800

[21] Au HD Lan NT Binh NT Linh Le Tuan Hien MM Duc NM. Sporadic Creutzfeldt-Jakob disease: brain MRI lesion features from 2 cases reports. Radiol Case Rep. 2024;19:939–43. 38188942 10.1016/j.radcr.2023.11.082 PMC10767313

[22] Li Z Li J Wang S Wang X Chen J Qin L. Laminar profile of auditory steady-state response in the auditory cortex of awake mice. Front Syst Neurosci. 2021;15:636395. 33815073 10.3389/fnsys.2021.636395 PMC8017131

[23] Manix M Kalakoti P Henry M. Creutzfeldt-Jakob disease: updated diagnostic criteria, treatment algorithm, and the utility of brain biopsy. Neurosurg Focus. 2015;39:E2. 10.3171/2015.8.FOCUS15328 26646926

[24] Qi C Zhang JT Zhao W Xing X-W Yu S-Y. Sporadic Creutzfeldt-Jakob disease: a retrospective analysis of 104 cases. Eur Neurol. 2020;83:65–72. 32344417 10.1159/000507189 PMC7265760

[25] Attaripour Isfahani S Dougherty M Gliebus GP. Applicability of long-term electroencephalography in pre-mortem diagnosis of Creutzfeldt-Jakob disease: a case report. SAGE Open Med Case Rep. 2017;5:2050313X17744482. 10.1177/2050313X17744482 PMC5734438 29276596

[26] Zerr I Schulz-Schaeffer WJ Giese A. Current clinical diagnosis in Creutzfeldt-Jakob disease: identification of uncommon variants. Ann Neurol. 2000;48:323–9. 10976638

[27] Balash Y Korczyn AD Khmelev N Eilam A Adi M Gilad R. Creutzfeldt-Jakob and vascular brain diseases: their overlap and relationships. Front Neurol. 2021;12:613991. 33732205 10.3389/fneur.2021.613991 PMC7959761

[28] Sanchez-Juan P Sánchez-Valle R Green A. Influence of timing on CSF tests value for Creutzfeldt-Jakob disease diagnosis. J Neurol. 2007;254:901–6. 17385081 10.1007/s00415-006-0472-9 PMC2779401

[29] Rafieiolhosseini N Killa M Neumann T. Computational model predicts protein binding sites of a luminescent ligand equipped with guanidiniocarbonyl-pyrrole groups. Beilstein J Org Chem. 2022;18:1322–31. 36225729 10.3762/bjoc.18.137 PMC9520824

[30] Bagyinszky E Giau VV Youn YC An SSA Kim SY. Characterization of mutations in PRNP (prion) gene and their possible roles in neurodegenerative diseases. Neuropsychiatr Dis Treat. 2018;14:2067–85. 30147320 10.2147/NDT.S165445 PMC6097508

[31] Baldwin KJ Correll CM. Prion disease. Semin Neurol. 2019;39:428–39. 31533183 10.1055/s-0039-1687841

[32] Jeong BH Kim YS. Genetic studies in human prion diseases. J Korean Med Sci. 2014;29:623–32. 24851016 10.3346/jkms.2014.29.5.623 PMC4024956

[33] Maynur G Yueying TAN. The value of diffusion-weighted MRI and EEG in the diagnosis of Creutzfeldt-Jakob disease. Xinjiang Med. 2007;37:79–81.

